# Supplementary material for: Cloning, Expression, Purification, and Characterization of β-Galactosidase from Bifidobacterium longum and Bifidobacterium pseudocatenulatum
Source: Molecules. 2022 Jul 14;27(14):4497. doi: 10.3390/molecules27144497 (PMC9323360; doi:10.3390/molecules27144497)
Supplement: Supplementary file 1 [file molecules-27-04497-s001.zip › Supplementary Figures.pdf]

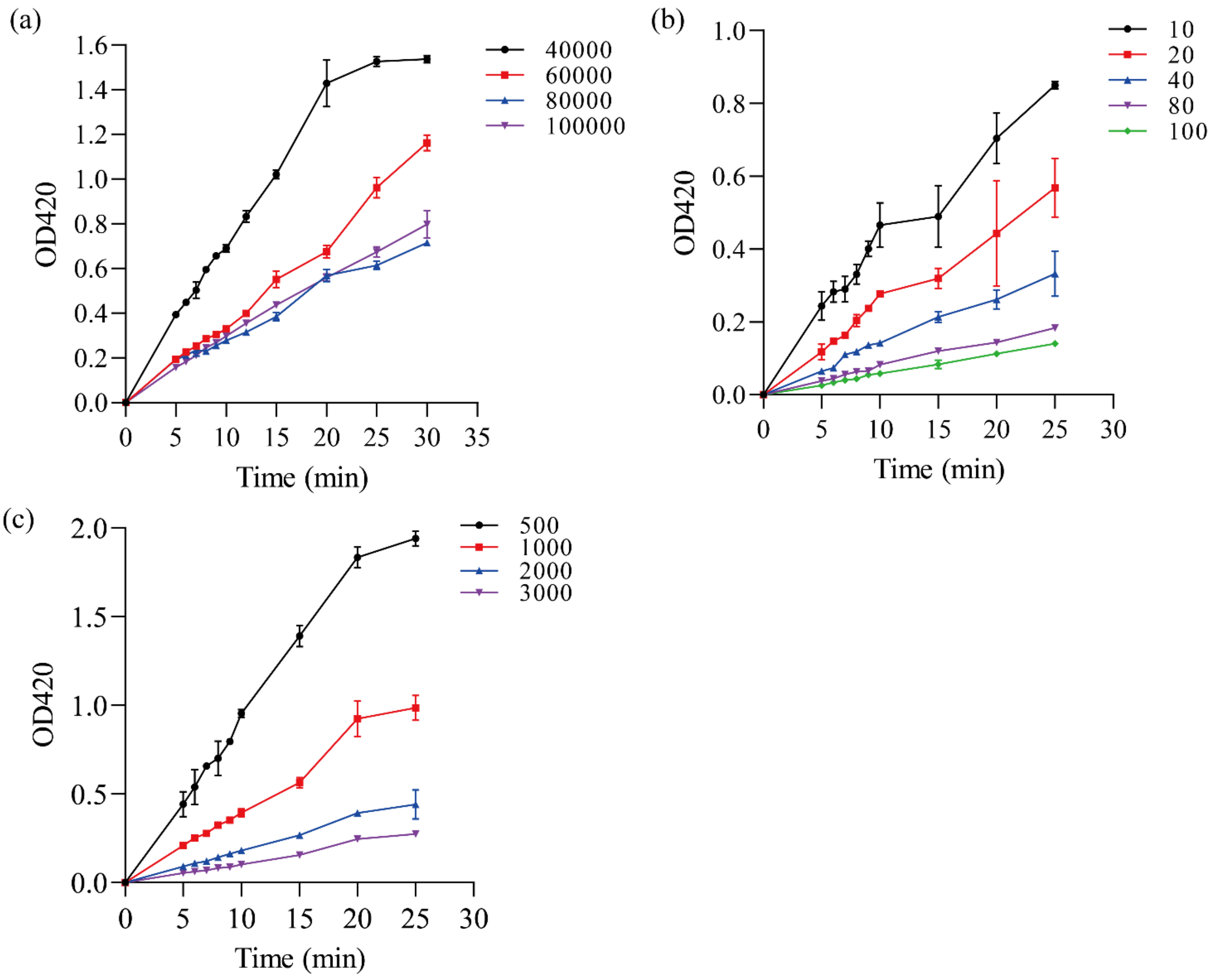

Figure S1. Enzyme reaction of BLGLB1 (a), BPGLB1 (b) and commercial β-galactosidase E (c) at different dilution gradients and reaction times. The numbers in the legend indicate the dilution factor， The initial concentration of BLGLB1, BPGLB1 and commercial β-galactosidase E were 34.8 mg/mL、17.8 mg/mL, and 21.92 mg/mL respectively.

(a)

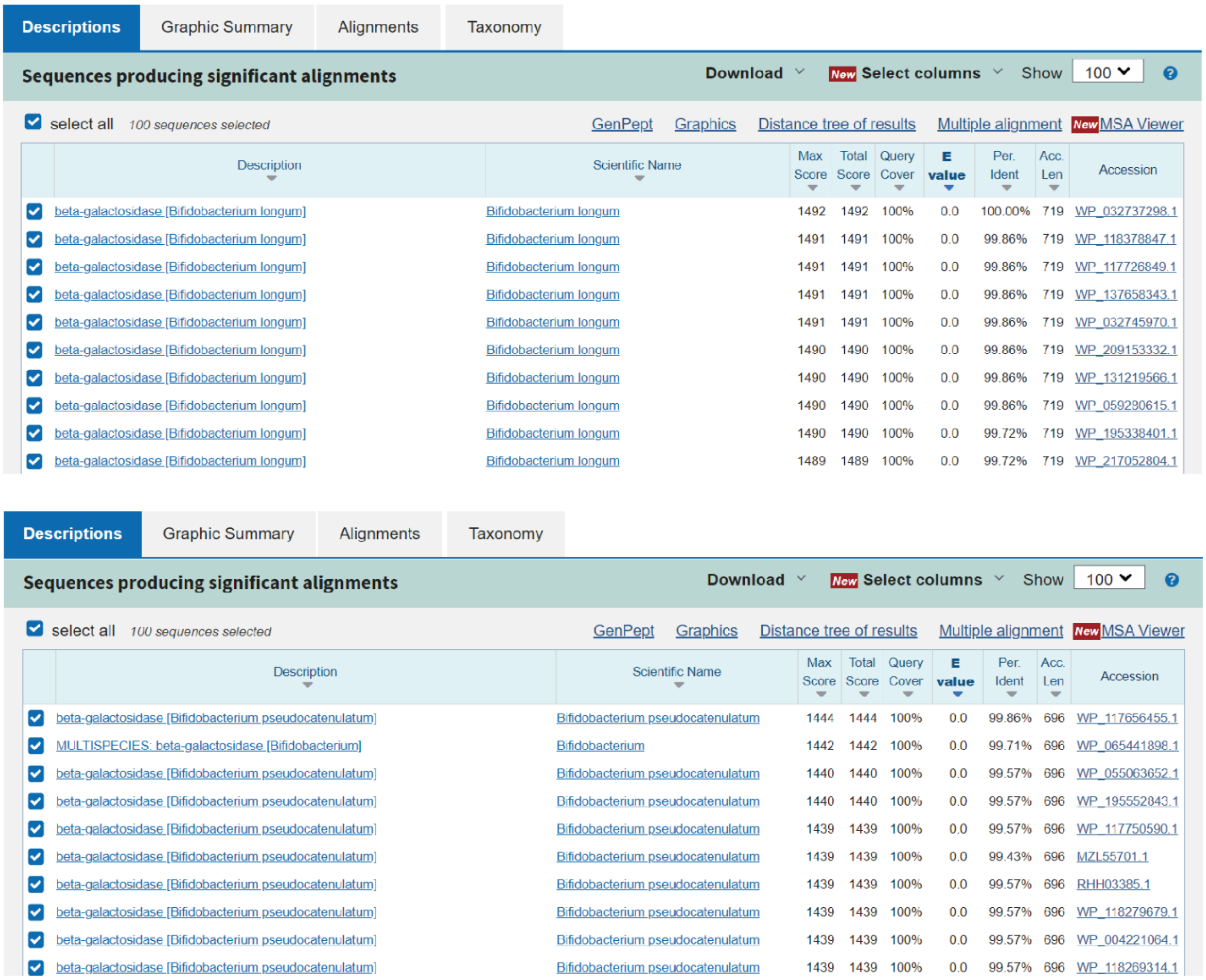

Descriptions | Graphic Summary | Alignments | Taxonomy

Sequences producing significant alignments — Download — New Select columns — Show 100

select all 100 sequences selected — GenPept Graphics Distance tree of results Multiple alignment New MSA Viewer

Description	Scientific Name	Max Score	Total Score	Query Cover	E value	Per. Ident	Acc. Len	Accession
beta-galactosidase [Bifidobacterium longum]	Bifidobacterium longum	1492	1492	100%	0.0	100.00%	719	WP_032737298.1
beta-galactosidase [Bifidobacterium longum]	Bifidobacterium longum	1491	1491	100%	0.0	99.86%	719	WP_118378847.1
beta-galactosidase [Bifidobacterium longum]	Bifidobacterium longum	1491	1491	100%	0.0	99.86%	719	WP_117726849.1
beta-galactosidase [Bifidobacterium longum]	Bifidobacterium longum	1491	1491	100%	0.0	99.86%	719	WP_137658343.1
beta-galactosidase [Bifidobacterium longum]	Bifidobacterium longum	1491	1491	100%	0.0	99.86%	719	WP_032745970.1
beta-galactosidase [Bifidobacterium longum]	Bifidobacterium longum	1490	1490	100%	0.0	99.86%	719	WP_209153332.1
beta-galactosidase [Bifidobacterium longum]	Bifidobacterium longum	1490	1490	100%	0.0	99.86%	719	WP_131219566.1
beta-galactosidase [Bifidobacterium longum]	Bifidobacterium longum	1490	1490	100%	0.0	99.86%	719	WP_059280615.1
beta-galactosidase [Bifidobacterium longum]	Bifidobacterium longum	1490	1490	100%	0.0	99.72%	719	WP_195338401.1
beta-galactosidase [Bifidobacterium longum]	Bifidobacterium longum	1489	1489	100%	0.0	99.72%	719	WP_217052804.1

(b)

Descriptions | Graphic Summary | Alignments | Taxonomy

Sequences producing significant alignments — Download — New Select columns — Show 100

select all 100 sequences selected — GenPept Graphics Distance tree of results Multiple alignment New MSA Viewer

Description	Scientific Name	Max Score	Total Score	Query Cover	E value	Per. Ident	Acc. Len	Accession
beta-galactosidase [Bifidobacterium pseudocatenulatum]	Bifidobacterium pseudocatenulatum	1444	1444	100%	0.0	99.86%	696	WP_117656455.1
MULTISPECIES: beta-galactosidase [Bifidobacterium]	Bifidobacterium	1442	1442	100%	0.0	99.71%	696	WP_065441898.1
beta-galactosidase [Bifidobacterium pseudocatenulatum]	Bifidobacterium pseudocatenulatum	1440	1440	100%	0.0	99.57%	696	WP_055063652.1
beta-galactosidase [Bifidobacterium pseudocatenulatum]	Bifidobacterium pseudocatenulatum	1440	1440	100%	0.0	99.57%	696	WP_195552843.1
beta-galactosidase [Bifidobacterium pseudocatenulatum]	Bifidobacterium pseudocatenulatum	1439	1439	100%	0.0	99.57%	696	WP_117750590.1
beta-galactosidase [Bifidobacterium pseudocatenulatum]	Bifidobacterium pseudocatenulatum	1439	1439	100%	0.0	99.43%	696	MZL55701.1
beta-galactosidase [Bifidobacterium pseudocatenulatum]	Bifidobacterium pseudocatenulatum	1439	1439	100%	0.0	99.57%	696	RHH03385.1
beta-galactosidase [Bifidobacterium pseudocatenulatum]	Bifidobacterium pseudocatenulatum	1439	1439	100%	0.0	99.57%	696	WP_118279679.1
beta-galactosidase [Bifidobacterium pseudocatenulatum]	Bifidobacterium pseudocatenulatum	1439	1439	100%	0.0	99.57%	696	WP_004221064.1
beta-galactosidase [Bifidobacterium pseudocatenulatum]	Bifidobacterium pseudocatenulatum	1439	1439	100%	0.0	99.57%	696	WP_118269314.1

Figure S2. NCBI BLASTp results of the BLGLB1 (a), BPGLB1 (b).

```
                                1        10        20        30        40        50
AAR24113.1   ..........MSARRNFEWPELLTADGRGIAFGGDYNPDQWSEDIWDDDIRLMKQAGVNT
ACJ53184.1   ..........MRARRDFAWPKLLTADGRGIAFGGDYNPDQWPEDVWDDDIRLMGQAGVNT
BPGLB1       ............MHRTFKWPELLTDNGKGIAYGGDYNPDQWSEDVWDEDVRLMKQAGVNT
ABE95118.1   ..........MTTRRAFRWPSLLTESGRGIAFGGDYNPDQWPEETLDEDIRLMGEAGVNV
BAR71185.1   ..........MTTRRAFRWPSLLTESGRGIAFGGDYNPDQWPEETLDEDIRLMDEAGVNV
ALO71994.1   ..........MTTHRAFRWPSLLTESGRGIAFGGDYNPDQWPEETLDEDIRLMGEAGVNV
BLGLB1       ..........MTTRRTFRWPSLLTESGRGIAFGGDYNPDQWSEETLDEDIRLMVQAGVNT
ACJ53083.1   ...........MEHRAFKWPQPLAGNKPRIWYGGDYNPDQWPEEVWDEDVALMQQAGVNL
AAL02053.1   ...........MEHRAFKWPQPLAGNKPRIWYVGDYNPDQWPEEVWDEDVALMQQAGVNL
AAU00556.1   ...........MEHREFKWPQPLAGGKPRIWYGGDYNPDQWPEEVWDEDVALMQKAGVNL
ABE95226.1   ...........MEHREFKWPQPLAGGKPRIWYGGDYNPDQWPEEVWDEDVALMQKAGVNL
ACD98372.1   ...........MERKEFKWPQPLAGNEPRIWYGGDYNPDQWPEEVWDEDIELMQQAGVNL
BAQ98551.1   ......MERNMSKRRKHSWPQPLKGAESRLWYGGDYNPDQWPEEVWDDDIRLMKKAGVNL
ABP87597.1   ..........MSKRRKHSWPQPLKGAESRLWYGGDYNPDQWPEEVWDDDIRLMKKAGVNL
ADO53518.1   ..........MSKRRKHSWPQPLKGAESRLWYGGDYNPDQWPEEVWDDDIRLMKKAGVNL
CAC14567.1   ..........MAQRRAHRWPKPLSGRPDRIWYGGDYNPDQWPEDVWDEDIRLMRQAGVNL
AGH68883.1   .......MSASTQHRAHRWPQPLPGNDRKIWFGADYNPDQWPEDVQDEDIRLMKQAGVNI
ACS45863.1   .......MSASTQHRAHRWPQPLPGNDRKIWFGADYNPDQWPEDVQDEDIRLMKQAGVNI
ACJ53470.1   MTDTMAHTQPASPASPASPAPTITAPGGRFVFGGDWNPEQWDESTWADDIAKLERAGINE
ACS45449.1   ............MARAYTDPILFG.....AAYYDEYIP..RDLDRIDTDMEMMTRAGINV

                       60        70        80        90       100       110
AAR24113.1   VALAIFSWDRIQPTEDRWDFGWLDRIIDKLGNAGIVVDLASATATAPLWLYESHPEVLPR
ACJ53184.1   VALAIFSWDRLQPTEDHWNFDWLDRIIDKLGQAGIAVDLASATATAPLWLYENHPEVLPR
BPGLB1       VAVAIFSWDRIQPEENRWDFGWLDRIIDKLGKAGIAVDLASATATAPLWLYEKHPEVLPQ
ABE95118.1   VSLAIFSWDKIEPVEGAFTFEWLDHVIDRLGKAGIAVDLASATATAPLWLYESHPEVLPV
BAR71185.1   VSLAIFSWDKIEPVEGAFTFEWLDHVIDRLGKAGIAVDLASATATAPLWLYESHPEVLPV
ALO71994.1   VSLAIFSWDKIEPVEGAFTFEWLDHVIDRLGRAGIAVDLASATAAAPLWLYESHPEVLPV
BLGLB1       VALAIFSWDKIEPREGEFTFEWLDHVIDKLGAAGIAVDLASATATAPLWLYERHPEVLPI
ACJ53083.1   VSVAIFSWAKLEPEEGVYDFDWLDRVIDKLGKAGIAVDLASGTASPPMWMTQAHPEILWV
AAL02053.1   VSVAIFSWAKLEPEEGVYDFDWLDRVIDKLGKAGIAVDLASGTASPPMWMTQAHPEILWV
AAU00556.1   VSVAIFSWAKLEPEEGVYDFDWLDRVIDKLGKAGIAVDLASGTASPPMWMTQAHPEILWV
ABE95226.1   VSVAIFSWAKLEPEEGVYDFDWLDRVIDKLGKAGIAVDLASGTASPPMWMTQAHPEILWV
ACD98372.1   VSVAIFSWAKLEPEEGVYDFDWLDRVIDKLGKAGIAVDLASGTASPPMWMTQAHPEILWV
BAQ98551.1   VSVGIFSWAKIEPEEGKYDFDWLDRAIDKLGKAGIAVDLASATASPPMWLTQAHPEVLWK
ABP87597.1   VSVGIFSWAKIEPEEGKYDFDWLDRAIDKLGKAGIAVDLASATASPPMWLTQAHPEVLWK
ADO53518.1   VSVGIFSWAKIEPEEGKYDFDWLDRAIDKLGKAGIAVDLASATASPPMWLTQAHPEVLWK
CAC14567.1   VSVGIFSWARIEPEEGVYDFGWLDRIIDKLGKAGIAVDLASATASPPMWLTQAHPEVLWK
AGH68883.1   VSLAIFSWANIETSDGNFEFDWLDRVIDKLYKAGIAVDLASATASPPMWLTSAHPEVLRR
ACS45863.1   VSLAIFSWANIETSDGNFEFDWLDRVIDKLYKAGIAVDLASATASPPMWLTSAHPEVLRR
ACJ53470.1   ATINVFSWALIQPDESRYDFAMLDRIVDLLVAHDFGFVLATSTGALPAWIAQRYPDATRT
ACS45449.1   IRIGESTWSTCEPQPGHFDWTHIDRALDAATNAGINVIVGTPTYAVPTWLVAMYPDVLAT

                     120       130       140       150       160       170
AAR24113.1   DKYGHPVNAGSRQSWSPTSPVFKEYALTLCRKLAERYGTNPYVTAWHMGNEYGWNNREDY
ACJ53184.1   DKYGHPVNAGSRQSWSPTSPVFKEYALTLCRKLAERYGTNPYVTAWHMGNEYGWNNRDDY
BPGLB1       DKFGHPVNAGSRQSWSPTSPVFKEYALTLCRKLAERYGTNPYVTAWHMGNEYGWNNRYDY
ABE95118.1   DRYGHIVNAGSRQSWQPTSPVFKEYALRLCRKLAERYKDNPYVTAWHMGNEYGWNNRYDY
BAR71185.1   DRYGHIVNAGSRQSWQPTSPVFKEYALRLCRKLAEHYKDNPYVTAWHMGNEYGWNNRYDY
ALO71994.1   DRYGHTVNAGSRQSWQPTSPVFKEYALRLCRRLAEHYKDNPYVTAWHMGNEYGWNNRYDY
BLGLB1       DRYGHVVNAGSRQSWQPTSPVLKEYALRLCRKLAEHYKDNPYVTAWHMGNEYGWNNRYDY
ACJ53083.1   DYRGDVCQPGARQHWRATSPVFLDYALNLCRKMAEHYKDNPYVVSWHVSNEYGCHNRFDY
AAL02053.1   DYRGDVCQPGARQHWRATSPVFLDYALNLCRKMAEHYKDNPYVVSWHVSNEYGCHNRFDY
AAU00556.1   DYRGDVCQPGARQHWRATSPVFLDYALNLCRKMAEHYKDNPYVVSWHVSNEYGCHNRFDY
ABE95226.1   DYRGDVCQPGARQHWRATSPVFLDYALNLCRKMAEHYKDNPYVVSWHVSNEYGCHNRFDY
ACD98372.1   DYRGDVCQPGARQHWRATSPVFLDYALNLCRKMAEHYKDNPYVVSWHVSNEYGCHNRFDY
BAQ98551.1   DERGDTVWPGAREHWRPTSPVFREYALNLCRRMAEHYKGNPYVVAWHVSNEYGCHNRFDY
ABP87597.1   DERGDTVWPGAREHWRPTSPVFREYALNLCRRMAEHYKGNPYVVAWHVSNEYGCHNRFDY
ADO53518.1   DERGDTVWPGAREHWRPTSPVFREYALNLCRRMAEHYKGNPYVVAWHVSNEYGCHNRFDY
CAC14567.1   DERGDVCQPGARQHWRPTSPVFREYALNLCRAMAEHYRDNPYVVAWHVSNEYGCHNRFDY
AGH68883.1   DEQGHVIWPGARQHWRPTSPTFRTYALRLCREMAEHYKDNPAIVSWHVGNEYGCHNYFDY
ACS45863.1   DEQGHVIWPGARQHWRPTSPTFRTYALRLCREMAEHYKDNPAIVSWHVGNEYGCHNYFDY
ACJ53470.1   DYEGRRHRFGVRHNACPNSPNFLRLAGALAGKLAERYGANDHLIAWHISNELGG...RCY
ACS45449.1   TPAG.EPHYGARQIMNIVNPAYRLYGERVIRSLISHVAQQPCVIGYQVDNETKYYDSVSH

                     180       190       200       210           220
AAR24113.1   SDNALEAFRAWCRRKYGTIDALNQAWGTTFWGQEMNGFDEVLHPTVHGR...RLRWSTPA
ACJ53184.1   SDNALEAFRAWCRRKYGTIDALNQAWGTTFWGQEMTGFDEVLIPRFMG....ADSMVNPG
BPGLB1       SDNALNAFRLWCERKYGTIENLNKAWGTTFWGQEMNGFHEVLIPRFMG....ADSMVNPG
ABE95118.1   SDNALAAFRTWCEAKYGTIDALNEAWGTAFWSQHVNSFDEVLLPRHMG....GDAMVNPP
BAR71185.1   SDNALAAFHTWCEAKYGTIDALNEAWGTAFWSQHVNSFDEVLLPRHMG....GDAMVNPP
ALO71994.1   SDNALAAFRTWCEAKYGTIDALNEAWGTAFWSQHVNSFDEVLLPRHMG....GDAMVNPS
BLGLB1       SDNALAAFRTWCEAKYGTVDALNEAWGTAFWSQHVNSFDEVLLPRHMG....GDSMVNPP
ACJ53083.1   SEDAERAFQKWCEKKYGTIDAVNDAWGTAFWAQRMNNFSEIIPPRFIG....DGNFMNPG
AAL02053.1   SEDAERAFQKWCEKKYGTIDAVNDAWGTAFWAQRMNNFSEIIPPRFIG....DGNFMNPG
AAU00556.1   SEDAERAFQKWCEKKYGTIDAVNDAWGTAFWAQRMNNFSEIIPPRFIG....DGNFMNPG
ABE95226.1   SEDAERAFQKWCEKKYGTIDAVNDAWGTAFWAQRMNNFSEIIPPRFIG....DGNFMNPG
ACD98372.1   SEDAERAFQKWCEKKYGTIDAVNDAWGTAFWAQRMNNFSEIIPPRFIG....DGNFMNPG
BAQ98551.1   SDDAMRAFQKWCKKRYKTIDAVNEAWGTAFWAQHMNDFSEIIPPRYIG....DGNFMNPG
ABP87597.1   SDDAMRAFQKWCKKRYKTIDAVNEAWGTAFWAQHMNDFSEIIPPRYIG....DGNFMNPG
ADO53518.1   SDDAMRAFQKWCKKRYKTIDAVNEAWGTAFWAQHMNDFSEIIPPRYIG....DGNFMNPG
CAC14567.1   SEDAERAFRQWCEERYGTIEKVNDAWGTAFWAHGMNDFSEIIPPRFIG....EGNFMNPG
AGH68883.1   SDDAVQAFREWCRDRYGTIDKVNAAWGTNFWSQRLNSFEEILPPRYVGG...EGNFTNPG
ACS45863.1   SDDAVQAFREWCRDRYGTIDKVNAAWGTNFWSQRLNSFEEILPPRYVGG...EGNFTNPG
ACJ53470.1   CDNCAAAFRVWLERKYGSIEALNRAWNANFWSHTYADFAQILPPNAISDGLDGERATLSA
ACS45449.1   DMQVMFIKQLRHEFKN.DLEALNEAYGLDYWSNRIN..AWEDFPDLTG.......SINES
```

```
              230       240       250       260              270       280
AAR24113.1   RSSTFERFGNDMLLDFYKAERDAIAEICPDKPFTTN.......FMVSTDQCCMDYAAWAK
ACJ53184.1   QKLDFERFGNDMLLDFYKAERDAIAEICPDKPFTTN.......FMISTDQCCMDYAAWAE
BPGLB1       QKLDFERFGNDMLLDFYKAERDAIAEICPDKPFTTN.......FMVSTDQCCMDYADWAE
ABE95118.1   QQLDYERFGNDMLLDFYKAERDAIEQICPGKPFTTN.......FMVSTDQCVMDYAKWAG
BAR71185.1   QQLDYERFGNDMLLDFYKAERDAIEQICPDKPFTTN.......FMVSTDQCVMDYAKWAG
ALO71994.1   QQLDYERFGNDMLLDFYKAERDAIEQICPDKPFTTN.......FMVSTDQCVMNYAKWAD
BLGLB1       QQLDYERFGNDMLLDFYKAERDAIEEICPGKPFTTN.......FMVSTDQCTMDYAQWAN
ACJ53083.1   KLLDWKRFSSDALLDFYKAERDALLEIAP.KPQTTN.......FMVSAGCTVLDYDKWGH
AAL02053.1   KLLDWKRFSSDALLDFYKAERDALLEIAP.KPQTTN.......FMVSAGCTVLDYDKWGH
AAU00556.1   KLLDWKRFSSDALLDFYKAERDALLEIAP.KPQTTN.......FMVSAGGAGIDYDKWGY
ABE95226.1   KLLDWKRFSSDALLDFYKAERDALLEIAP.KPQTTN.......FMVSAGGAGIDYDKWGY
ACD98372.1   KLLDWKRFSSDALLDFYKAERDALLEIAP.KPQTTN.......FMVSAGGTGIDYDKWGY
BAQ98551.1   KLLDYKRFSSDALKELYIAERDVLESITPGLPLTTN.......FMVSAGGSMLDYDDWGA
ABP87597.1   KLLDYKRFSSDALKELYIAERDVLESITPGLPLTTN.......FMVSAGGSMLDYDDWGA
ADO53518.1   KLLDYKRFSSDALKELYIAERDVLESITPGLPLTTN.......FMVSAGGSMLDYDDWGA
CAC14567.1   KLLDFKRFSSDALKAFYMAEPDTLAEITPDLPLTTN.......FMVSASGTGLDYDDWGG
AGH68883.1   RLLDFKHFCSDALKEFFCAERDVLSEVTPNIPLTTN.......FMVSASQNTLDYDDWAH
ACS45863.1   RLLDFKHFCSDALKEFFCAERDVLSEVTPNIPLTTN.......FMVSASQNTLDYDDWAH
ACJ53470.1   CSIDYKRFQSDSLLGTYVTERDAIRAFDAMHPITTN.......LMDTYEG..ADYFRWGR
ACS45449.1   LRARFDRFRRDQVAEYLAWQASIIREYMRDDQFITHNFDYEWRGHSYGLQPAVDHFRAAR
```

```
                     290       300       310       320       330
AAR24113.1   EVNFVSNDHYFHEGESHLD.ELACSDALMDSLALGKPWYVMEHSTSAVQWKPLNTRKRKG
ACJ53184.1   EVNFVSNDHYFHEGESHLD.ELACSDALVDSLALGKPWYVMEHSTSAVQWKPLNTRKRNG
BPGLB1       EVDFVSNDHYFHEGESHID.ELFCSDALMDSLALGKPWYVMEHSTSAVQWKPLNARKRKG
ABE95118.1   EVDFVSNDHYFHEGESHLD.ELACSDALMDSLALGKPWYVMEHSTSAVQWKPLNARKRAG
BAR71185.1   EVDFVSNDHYFHEGESHLD.ELACSDALMDSLALGKPWYVMEHSTSAVQWKPLNARKRAG
ALO71994.1   EVDFVSNDHYFHEGESHLD.ELACSDALMDSLALGKPWYVMEHSTSAVQWKPLNTRKRAG
BLGLB1       EVDFVSNDHYFHEGESHLD.ELACSDALMDSLALGKPWYVMEHSTSAVQWKPLNMRKRAG
ACJ53083.1   DVDFVSNDHYFSPGEAHFD.EMAYAACLTDGIARKNPWFLMEHSTSAVNWRPTNYRLEPG
AAL02053.1   DVDFVSNDHYFSPGEAHFD.EMAYAACLTDGIARKNPWFLMEHSTSAVNWRPTNYRLEPG
AAU00556.1   DVDFVSNDHYFTPGEAHFD.ELAYSASLCDGIARKNPWFLMEHSSSAVNWRPINYRVEPG
ABE95226.1   DVDFVSNDHYFTPGEAHFD.ELAYSASLCDGIARKNPWFLMEHSSSAVNWRPINYRVEPG
ACD98372.1   DVDFVSNDHYFTPGEAHFD.ELAYSASLCDGIARKNPWFLMEHSSSAVNWRPINYRVEPG
BAQ98551.1   EVDFVSNDHYFTPGEDHFD.EVAYAASLMDGISRKEPWFQMEHSTSAVNWRPINYRAEPG
ABP87597.1   EVDFVSNDHYFTPGEDHFD.EVAYAASLMDGISRKEPWFQMEHSTSAVNWRPINYRAEPG
ADO53518.1   EVDFVSNDHYFTPGEDHFD.EVAYAASLMDGISRKEPWFQMEHSTSAVNWRPINYRAEPG
CAC14567.1   EVDFVSNDHYFTPGEAHLD.ELAFSASLVDGISRKNPWFLMEHSTSAVNWRPINYRKEPG
AGH68883.1   EVDFVSNDHYFTPGSWHID.ELAYSASLVDGISRKKPWFLMEQSTSAVNWREINPRKEPG
ACS45863.1   EVDFVSNDHYFTPGSWHID.ELAYSASLVDGISRKKPWFLMEQSTSAVNWREINPRKEPG
ACJ53470.1   EMDVISWDDYPFP.HTTPS.DNAFKHDLMRGVGDGRPFMLMESTPNQTNWQECNVLRAPG
ACS45449.1   ALDICGVDIYHPSEDALTGKEIAFGGDMARSAGGGNYLVLETQAQGQHGWLPY.....PG
```

```
            340       350       360       370       380       390
AAR24113.1   ETVRDSLAHVAMGADAINFFQWRASAFGAEAFHSAMVPHAG....EDTKLFRQVCELGAS
ACJ53184.1   ETVRDSLAHVAMGADAINFFQWRASAFGAEAFHSAMVPHAG....ENTKLFRQVCELGAT
BPGLB1       ETVRDSIAHVAMGADAINFFQWRASAFGAESFHSALVPHAG....EDTKLFRQVCELGAA
ABE95118.1   ELMRDSLAHVAMGADAICFFQWRQSVSGAEAFHSAMLPHAG....ADTKVFRGVCELGKA
BAR71185.1   ELMRDSLAHVAMGADAICFFQWRQSVSGAEAFHSAMLPHAG....ADSKVFRGVCELGKA
ALO71994.1   ELMRDSLAHVAMGADAICFFQWRQSKSGAEAFHSAMLPHAG....ADSKVFRGVCELGKA
BLGLB1       ELMRDSLAHVAMGADAINFFQWRQSASGAEAFHSAMVPHAG....SDTKLFRGVCELGAA
ACJ53083.1   ELVRDSLAHLAMGADAICYFQWRQSKAGAEKWHSAMVPHAG....PDSQIFRDVCELGAD
AAL02053.1   ELVRDSLAHLAMGADAICYFQWRQSKAGAEKWHSAMVPHAG....PDSQIFRDVCELGAD
AAU00556.1   ELVRDSLAHLAMGADAICYFQWRQSKAGAEKWHSAMVPHAG....PDSQIFRDVCELGAD
ABE95226.1   ELVRDSLAHLAMGADAICYFQWRQSKAGAEKWHSSMVPHAG....PDSQIFRDVCELGAD
ACD98372.1   ELVRDSLAHLAMGSDAICYFQWRQSKAGAEKWHSSMVPHAG....PDSQIFRDVCELGAD
BAQ98551.1   SVVRDSLAQVAMGADAICYFQWRQSKAGAEKWHSSMVPHAG....EDSQIFRDVCELGAD
ABP87597.1   SVVRDSLAQVAMGADAICYFQWRQSKAGAEKWHSSMVPHAG....EDSQIFRDVCELGAD
ADO53518.1   SVVRDSLAQVAMGADAICYFQWRQSKAGAEKWHSSMVPHAG....EDSQIFRDVCELGAD
CAC14567.1   QLVRDSLAHVAMGSDAVCYFQWRQSKAGAEKFHSAMLPHAG....EDSQVFRDVCELGSG
AGH68883.1   ELIRDSMLHLAMGADAICYFQWRQSRSGAEKFHSAMLPLAG....EHSQIYRDVCALGAD
ACS45863.1   ELIRDSMLHLAMGADAICYFQWRQSRSGAEKFHSAMLPLAG....EHSQIYRDVCALGAD
ACJ53470.1   RMRAESYQAVAHGADTVQYFQLKQSRGGFEKYHGAVISHGGR...EDERVYGEVRALGGE
ACS45449.1   QLRLQAYSHLASGADGIMYWHWHSIHNSFETYWRGLLSHDFESNPTYEEAGRFGREIGDP
```

```
                400       410       420       430       440       450
AAR24113.1   LHTLADAGVQGTELAHSDTAILFSAESEWATRSQTLPSMKLNHWHDVRD.WYRAFLDAGS
ACJ53184.1   LQALADAGVQGSELAHADTAILFSAESEWATRSETLPSMKLNHWHDVRD.WYRAFLNAGA
BPGLB1       LKTLGDAGVQGTELEQSDTAILFSAESEWATRSETLPSMKLNHWHDVRD.WYRAFLNAGT
ABE95118.1   LKTLSDAGLQGTELERAGTAILFSAESEWATRSETLPSMKLNHWHDVRD.WYRGFLDAGL
BAR71185.1   LKTLSDAGLQGTELERAGTAILFSAESEWATRSETLPSMKLNHWHDVRD.WYRGFLDAGL
ALO71994.1   LKTLSDAGLQGTELERAGTAILFSAESEWATRSETLPSMKLNHWHDVRD.WYRGFLDAGL
BLGLB1       LKTLSDAGVQDTELKRADTAILFSAESEWATRSETLPSMKLNHWHDVRD.WYRGYLDAGA
ACJ53083.1   LNKLADEGLLSTKLVKSKVAIVFDYESQWATEHTATPTQEVRHWTEPLD.WFRALADNGL
AAL02053.1   LNKLADEGLLSTKLVKSKVAVVFDYESQWATEHTATPTQEVRHWTEPLD.WFRALADNGL
AAU00556.1   LNKLADEGLLSTKLVKSKVAVVFDYESQWATEHTATPTQEVRHWTEPLA.WFRALADNGL
ABE95226.1   LNKLADEGLLSTKLVKSKVAVVFDYESQWATEHTATPTQEVRHWTEPLA.WFRALADNGL
ACD98372.1   LNKLADEGLLSTKLVKSKVAVVFDYESQWATEHTATPTQEVRHWTEPLA.WFRALADNGL
BAQ98551.1   LGRLSDEGLMGTKTVKSKVAVVFDYESQWATEYTANPTQQVDHWTEPLD.WFRALADNGI
ABP87597.1   LGRLSDEGLMGTKTVKSKVAVVFDYESQWATEYTANPTQQVDHWTEPLD.WFRALADNGI
ADO53518.1   LGRLSDEGLMGTKTVKSKVAVVFDYESQWATEYTANPTQQVDHWTEPLD.WFRALADNGI
CAC14567.1   LNALADNGLLGTRLAKSRVAVVYDYESEWASEHTATPTQKVRHVDEPLA.WFRALADNGV
AGH68883.1   LDTLSDAGILRSKLSKARVAIVQDIQSEWATEHTATPTQHIREWTEPLD.WFAAFANRGV
ACS45863.1   LDTLSDAGILRSKLSKARVAIVQDIQSEWATEHTATPTQHIREWTEPLD.WFAAFANRGV
ACJ53470.1   LAAHGARFVGG..LTEAPVALMFDWDSYWSTENISLLPKGFDYPDQVRR.WYAPFHHRNI
ACS45449.1   RIGDTLSHLSKRNAVAILASNESLTALSWFHIETGFPMGGTLTYNDVLRSIYDALFELNV
```

```
                   460       470       480       490       500       510
AAR24113.1   RADIVPLAYDWS...SYKTVVLPTVLILSAADTQRLADFAAAGGRVVVGYATGLIDEHFH
ACJ53184.1   RADIVPLAYDWS...SYKTIVLPTVLMLSDADTRRLAGFVQDGGRVVVGYATGLLDERFH
BPGLB1       RADIVPLKYDWS...AYKTVVLPTVIMLSAEDTQRLADFAAAGGRVVIGYATGLIDENFH
ABE95118.1   RADVVPLAYDWT...DYKTIVLPTVLSLSDEDVRRIADFAKAGGTVIVGYATGLVDEYFH
BAR71185.1   RADVVPLAYDWT...DYKTIVLPTVLSLPDEDVRRIADFAKAGGTVIVGYATGLIDEHFH
ALO71994.1   RADVVPLAYDWT...GYKTIVLPTVLSLSDEDVLRIADFAKAGGTVIVGYAAGLIDEHFH
BLGLB1       RADVVPLAYDWS...GYQTIVLPTVIALSDEDTRRIADFAENGGTVIVGYATGLIDEHFH
ACJ53083.1   TADVVPVRGPWD...EYEAVVLPSLAILSEQTTRRVREYVANGGKLFVTYYTGLVDDRDH
AAL02053.1   TADVVPVRGPWD...EYEAVVLPSLAILSEQTTRRVREYVANGGKLFVTYYTGLVDDRDH
AAU00556.1   TADVVPVRGPWD...EYEAVVLPSLTILSEETTRRVREYVANGGKLFVTYYTGLVDDKDH
ABE95226.1   TADVVPVRGPWD...EYEAVVLPSLTILSEETTRRVREYVANGGKLFVTYYTGLVDDKDH
ACD98372.1   TADVVPVRGPWD...EYEAVVLPSLTILSEETTRRVREYVANGGKLFVTYYTGLVDDKDH
BAQ98551.1   TADVVPVRSDWD...SYEIAVLPCVYLLSEETSRRVREFVANGGKLFVTYYTGLSDENDH
ABP87597.1   TADVVPVRSDWD...SYEIAVLPCVYLLSEETSRRVREFVANGGKLFVTYYTGLSDENDH
ADO53518.1   TADVVPVRSDWD...SYEIAVLPCVYLLSEETSRRVREFVANGGKLFVTYYTGLSDENDH
CAC14567.1   TADVVPVRSNWD...EYEVAVLPSVYILSEETTRRVREYVAGGGRLIVTYYTGLSDEKDH
AGH68883.1   TADVTPIHAQWD...TYDAVVIPCVYLFSEEMAERLRTFVRNGGKAFVTYYSALADEHDR
ACS45863.1   TADVTPIHAQWD...TYDAVVIPCVYLFSEEMAERLRTFVRNGGKAFVTYYSALADEHDR
ACJ53470.1   AVDVVPEDIDAGRLAGYRVLVAPALMMAKPGVRELVEGFVRAGGTFLATVMAGMHDEHDN
ACS45449.1   EVDFLPADASADQLAGYSLVIAPALYTTDQQTIDRLARYVKNGGHLLATMRSFVADENVK

                      520       530       540       550       560       570
AAR24113.1   TWLGGYPGAGDGLLRSMLGVRGEEFTILGAEAEGEPGEIRLSSADDSAALDGTTTRLWQN
ACJ53184.1   TWLGGYPGAGDGLLRSMLGVRGEEFNILGTETEDEPSEIRLASTGDSPTMDGAVTRLWQN
BPGLB1       TWLGGYPGAGDGLLREMLGIRGEEFNILGAEAEGEPSEIRLSSG........AVTRLWQN
ABE95118.1   IGLGGYPGAGNGLLRDMLGIRSEEFNILGEEAEGEPSEISLSNG........LTTRLWQN
BAR71185.1   IGLGGYPGAGNGLLRDMLGIRSEEFNILGEEAEGEPSEISLSNG........LMTRLWQN
ALO71994.1   IGLGGYPGAGNGLLRDMLGIRSEEFNILGEEAEGEPSEISLSNG........LTTRLWQN
BLGLB1       IGLGGYPGAGNGLLRDMLGIRSEEFNILGEEAEDEPAEIGLSNG........LTTRLWQN
ACJ53083.1   VWLGGYP....GSIRDVVGVRVEEFAPMGTDAPGTMDHLDLDNG........TVAHDFAD
AAL02053.1   VWLGGYP....GSIRDVVGVRVEEFAPMGTDAPGTMDHLDLDNG........TVAHDFAD
AAU00556.1   VWLGGYP....GSIRDVVGVRVEEFAPMGNDFPGAMDHLDLDNG........TVAHDFAD
ABE95226.1   VWLGGYP....GSIRDVVGVRVEEFAPMGNDFPGAMDHLDLDNG........TVAHDFAD
ACD98372.1   VWLGGYP....GSIRDVVGVRVEEFAPMGNDFPGAMDHLDLDNG........TVAHDFAD
BAQ98551.1   IWLGGYP....GSIRDVVGVRVEEFAPMGNDMPGALDHLDLDNG........TVAHDFAD
ABP87597.1   IWLGGYP....GSIRDVVGVRVEEFAPMGNDMPGALDHLDLDNG........TVAHDFAD
ADO53518.1   IWLGGYP....GSIRDVVGVRVEEFAPMGNDMPGALDHLDLDNG........TVAHDFAD
CAC14567.1   IWLGGYP....GSIRDVVGVRVEEFAPLGDDWPGIPEYLDLDNG........TTAHDFAD
AGH68883.1   LHTEGWP....GLIGDVVGVRIEEHCPLGTLFPGMLDHLDVSNG........TVVHDLAD
ACS45863.1   LHTEGWP....GLIGDVVGVRIEEHCPLGTLFPGMLDHLDVSNG........TVVHDLAD
ACJ53470.1   VILGGYP....GAFREVCGMRMEEMDMIPDGRDVRVVFGSGEGED.......ADTDGSRV
ACS45449.1   VWHDKAP....HHLVDIFGMTYNQFTRPMGVSLKCPDTLADLAG..............AS

                      580       590       600       610       620       630
AAR24113.1   DVNVTGEHAQVLATYAGEEADEWELDGTAAVTRNPYGSGEAYFVGCDLDVADLTKLVRAY
ACJ53184.1   DVTVAGPHVQVLAAYAGEEANEWELDGTAAITRNTYGEGEAYFLGCDLGVSDLTRFVGGW
BPGLB1       DVNVDGERAQVLATYEGEEADEWELGGAAAITRNPYGSGETYFVGCDLNVADLTEFVREN
ABE95118.1   DVTSVAADTTVLASYTGESAADWELERTPAITSHPYGNGTAIYVGCDLNRHDIAQLLKAL
BAR71185.1   DVTSVAADATVLASYTGESAADWELERTPAITSRPYGNGTAIYVGCDLNRHDIAQLLKAL
ALO71994.1   DVTSVAADTTVLASYAGKSAADWELERTPAITSRPYGNGTAIYVGCDLNRHDIAQLLKAL
BLGLB1       DVTSVAPDTRVLATYVGTAAADWELDGVPAITSHPHGQGAAIYVGCDLGRHDITHLLKEL
ACJ53083.1   VITSVADTAHVVASFKAD..KWTGFDGAPAITVNDFGDGKAAYVGARLGREGLAKSLPAL
AAL02053.1   VITSVADTAHVVASFKAD..KWTGFDGAPAITVNDFGDGKAAYVGARLGREGLAKSLPAL
AAU00556.1   VITSVADTAHVVAAFKAD..KWTGFDGAPAITINDFGDGKAAYVGARLGREGLAKSLPAL
ABE95226.1   VITSVADTAHVVASFKAD..KWTGFDGAPAITINDFGDGKAAYVGARLGREGLAKSLPAL
ACD98372.1   VITSVADTAHVVASFKAD..KWTGFDGAPAITVNDFGDGKAAYVGARLGREGLAKSLPVL
BAQ98551.1   VITSTADTSTVLASYKAE..RWTGMNEVPAIVANGYGDGRTVYVGCRLGRQGLAKSLPAM
ABP87597.1   VITSTADTSTVLASYKAE..RWTGMNEVPAIVANGYGDGRTVYVGCRLGRQGLAKSLPAM
ADO53518.1   VITSTADTSTVLASYKAE..RWTGMNEVPAIVANGYGDGRTVYVGCRLGRQGLAKSLPAM
CAC14567.1   VITSVGEHATVIASFKDD..PWTGMDGRPAIVGNAYGEGRTVYVGARLGRDGIARSLPMM
AGH68883.1   VIDAIADDTTVLATFEAD..PATGMDGRAAITVHPYHEGGVAYIAGKLGRDGISQSLPEI
ACS45863.1   VIDAIADDTTVLATFEAD..PATGMDGRAAITVHPYHEGGVAYIAGKLGRDGISQSLPEI
ACJ53470.1   SLVAGLIKLDGGARPLAAYAGDVFYRGTPAVTVNDFGAGTAYFAGAVLDEAGMDAVVGDV
ACS45449.1   ANDFIEMLSPAPETHVLAWYDHYAWDSYAAITRHAFGSGDAQWVGTQLQADAWRTVLAEA

                                                 640       650
AAR24113.1   LAASSQE..............................NADVLHTVRASAD..ATFDFYLP
ACJ53184.1   LAARPQDGRQPEA...............NLRSPASGVTSDVLHTVRQSDD..AIFDFYLT
BPGLB1       IVEDSAN.........................VANPTDSDVLHTVRKSAD..ATFDFYLS
ABE95118.1   GSRWREL.....SAQPTE.........GGQTPTYPTTDPRILHTIRRSADGSTRFDFYLN
BAR71185.1   GSRWREL.....SAQPTE.........GGQTPTYPTTDPRILHTIRRSADGSTRFDFYLN
ALO71994.1   GSRWQEL.....SAQPTE.........SGQTPTYPTTDPRILHTIRRSADGSTRFDFYLN
BLGLB1       NTTAPSD.....ERAPDQRPGGGEINAATTTAAATTHDPRILHTIRQSSDGTIRFDFYLN
ACJ53083.1   LEELGIETS........................AEDDRGEVLRVERADETGENHFVFLFN
AAL02053.1   LEELGIETS........................AEDDRGEVVRVERADETGENHFVFLFN
AAU00556.1   LEELGIETS........................AEDDRGEVLRVERADETGENHFVFLFN
ABE95226.1   LEELGIETS........................AEDDRGEVLRVERADETGENHFVFLFN
ACD98372.1   LEELGIETS........................AEDDRGEVLRVERADETGENHFVFLFN
BAQ98551.1   LGSMGLSDL........................AGD..GRVLRVERADAAAASRFEFVFN
ABP87597.1   LGSMGLSDL........................AGD..GRVLRVERADAAAASRFEFVFN
ADO53518.1   LGSMGLSDL........................AGD..GRVLRVERADAAAASHFEFVFN
CAC14567.1   FSSLGVEVP........................EASD.PGLLRVERVDEASGARFVFLFN
AGH68883.1   CAALGFELD........................ADPRAGDVLRVVREQED.GAIFEFLFN
ACS45863.1   CAALGFELD........................ADPRAGDVLRVVREQED.GAIFEFLFN
ACJ53470.1   VRRAGVTGIVS..........................PEPVEVVTRRYPSRGESLTFVIN
ACS45449.1   LSNAGVHTPG..............MELAGTVCVRSGTNTAGDTVTYLLNYSGSPITFRAP
```

```
              660       670       680       690       700
                .         .         .         .         .
AAR24113.1    RGKKTVELQGIEGEPVILFQTDREEKPGSYTVRRNGVLVVRR......
ACJ53184.1    RGKSDVELRDIAGEPIVLFRAERGSDGGAYTVHRNGVLVMKRPNPSV.
BPGLB1        RGKKEIALQGIEGEPIYLFQAEAEEQTGSYTVHRNGVLVVKRA.....
ABE95118.1    RSNQPVAINGVEGKPIIAHRCETDAVG..YTLNRNAILIAKTSC....
BAR71185.1    RSNQPVAINGVEGKPIIAHRCETDAVG..YTLNRNAILIAKTSC....
ALO71994.1    RSNQPVAINGVEGDPIIAHRCETDAVG..YTLNRNAILIAKTSC....
BLGLB1        RSKQPVAVNGVEGDPIIAHRCETDAVG..YTLNRNAILIAKTSC....
ACJ53083.1    R.THDVAVVDVEGEPLVASLAQVNESEHTAAIQPNGVLVVKL......
AAL02053.1    R.THDVAVVDVEGEPLVASLAQVNESEHTAAIQPNGVLVVKL......
AAU00556.1    R.THDVAIVDVDGEPLVASLAQVNESEHTAAIQPNGVLVVKL......
ABE95226.1    R.THDVAIVDVDGEPLVASLAQVNESEHTAAIQPNGVLVVKL......
ACD98372.1    R.THDVAVVDVEGEPLVASLAQVNESEHTAAIQPNGVLVVKL......
BAQ98551.1    R.THEPVTVDVEGEAIAASLAHVDDG..RATIDPTGVVVLRR......
ABP87597.1    R.THEPVTVDVEGEAIAASLAHVDDG..RATIDPTGVVVLRR......
ADO53518.1    R.THEPVTVDVEGEAIAASLAHVDDG..RATIDPTGVVVLRR......
CAC14567.1    R.THAVIEAMQDGRPVIASLADVGDG..KVTVRPNGVIVLRK......
AGH68883.1    R.TRNTVTADRPAG.DMLICSLATDSTDKVTLEPNGVLAFRR......
ACS45863.1    R.TRNTVTADRPAG.DMLICSLATDSTDKVTLEPNGVLAFRR......
ACJ53470.1    HADTATAWQDTPFAGCESVLDG.TVLGRDLVLEPYGVTVVRTAA....
ACS45449.1    ASGTFLLGHPTDDGEQAVTAETPVTVGDAVTLPRWGVDIIVGRQPTMN
```

Figure S3. a series of conserved amino acid residues of the BLGLB1 and BPGLB1.

```
             1        10        20        30        40        50        60
BLGLB1       MTTRRTFRWPSLLTESGRGIAFGGDYNPDQWSEETLDEDIRLMVQAGVNTVALAIFSWDK
BPGLB1       ..MHRTFKWPELLTDNGKGIAYGGDYNPDQWSEDVWDEDVRLMKQAGVNTVAVAIFSWDR
ABI35985.1   ....................MLGVCYYPEHWPKERWKEDARRMREAGLSHVRIGEFAWAL

                      70        80        90       100       110       120
BLGLB1       IEPREGEFTFEWLDHVIDKLGAAGIAVDLASATATAPLWLYERHPEVLPIDRYGHVVNAG
BPGLB1       IQPEENRWDFGWLDRIIDKLGKAGIAVDLASATATAPLWLYEKHPEVLPQDKFGHPVNAG
ABI35985.1   LEPEPGRLEWGWLDEAIATLAAEGLKVVLGTPTATPPKWLVDRYPEILPVDREGRRRRFG

                    130       140       150       160       170
BLGLB1       SRQSWQPTSPVLKEYALRLCRKLAEHYKDNPYVTAWHMGNEYGWNN.RYDYSDNALAAFR
BPGLB1       SRQSWSPTSPVFKEYALTLCRKLAERYGTNPYVTAWHMGNEYGWNN.RYDYSDNALNAFR
ABI35985.1   GRRHYCFSSPVYREEARRIVTLLAERYGGLEAVAGFQTDNEYGCHDTVRCYCPRCQEAFR

            180       190       200       210       220       230
BLGLB1       TWCEAKYGTVDALNEAWGTAFWSQHVNSFDEVLLPRHMGGDSMVNPPQQLDYERFGNDML
BPGLB1       LWCERKYGTIENLNKAWGTTFWGQEMNGFHEVLIPRFMGADSMVNPGQKLDFERFGNDML
ABI35985.1   GWLEARYGTIEALNEAWGTAFWSQRYRSFAEVELPHLTVAE..PNPSHLLDYYRFASDQV

            240       250       260       270       280
BLGLB1       LDFYKAERDAIEEICPGKPFTTNFMVSTDQCTMDYAQWANEVDFVSNDHY..........
BPGLB1       LDFYKAERDAIAEICPDKPFTTNFMVSTDQCCMDYADWAEEVDFVSNDHY..........
ABI35985.1   RAFNRLQVEILRAHAPGKFVTHNFMG..FFTDLDAFALAQDLDFASWDSYPLGFTDLMPL

                    290       300       310       320       330       340
BLGLB1       ......FHEGESHLDELACSDALMDSLALGKPWYVMEHSTSAVQWKPLNMRKRAGELMRD
BPGLB1       ......FHEGESHIDELFCSDALMDSLALGKPWYVMEHSTSAVQWKPLNARKRKGETVRD
ABI35985.1   PPEEKLRYARTGHPDVAAFHHDLYRGVGRGR.FWVMEQQPGPVNWAPHNPSPAPGMVRLW

                    350       360       370       380       390       400
BLGLB1       SLAHVAMGADAINFFQWRQSASGAEAFHSAMVPHAGSDTKLFRGVCELGAALKTLSDAGV
BPGLB1       SIAHVAMGADAINFFQWRASAFGAESFHSALVPHAGEDTKLFRQVCELGAALKTLGDAGV
ABI35985.1   TWEALAHGAEVVSYFRWRQAPFAQEQMHAGLHRPDSAPDQGFFEAKRVAEELAALALP..

                    410       420       430       440       450       460
BLGLB1       QDTELKRADTAILFSAESEWATRSETLPSMKLNHWHDVRDWYRGYLDAGARADVVPLAYD
BPGLB1       QGTELEQSDTAILFSAESEWATRSETLPSMKLNHWHDVRDWYRAFLNAGTRADIVPLKYD
ABI35985.1   ...PVAQAPVALVFDYEAAWIYEVQPQ.GAEWSYLGLVYLFYSALRRLGLDVDVVPPGAS

                    470       480       490       500       510       520
BLGLB1       WSGYQTIVLPTVIALSDEDTRRIADFAENGGTVIVGYATGLIDEHFHIGLGGYPGAGNGL
BPGLB1       WSAYKTVVLPTVIMLSAEDTQRLADFAAAGGRVVIGYATGLIDENFHTWLGGYPGAGDGL
ABI35985.1   LRGYAFAVVPSLPIVR...EEALEAFREAEGPVLFGPRSGSKTETFQIPKELPPGPLQAL

                    530       540       550       560       570       580
BLGLB1       LRDMLGIRSEEFNILGEEAEDEPAEIGLSNGLTTRLWQNDVTSVAPDTRVLATYVGTAAA
BPGLB1       LREMLGIRGEEFNILGAEAEGEPSEIRLSSGAVTRLWQNDVNVDGERAQVLATYEGEEAD
ABI35985.1   LPLKVVRVESLPPGLLEVAEGALGRFPLG......LWR......................

                    590       600       610       620       630       640
BLGLB1       DWELDGVPAITSHPHGQGAAIYVGCDLGRHDITHLLKELNTTAPSDERAPDQRPGGGEIN
BPGLB1       EWELGGAAAITRNPYGSGETYFVGCDLNVADLTEFVREN....................I
ABI35985.1   EWVEAPLKPLLTFQDGKGALYREGRYLYLAAWPSPELAG.....................
```

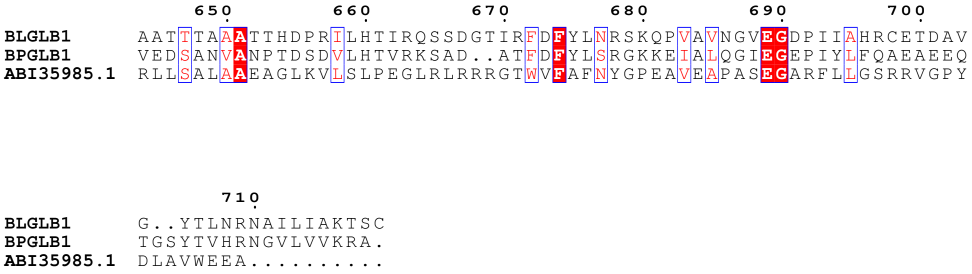

Figure S4. The key amino acid residue positions of sequence alignment of BLGLB1 and BPGLB1 with the β-galactosidase from *Thermus thermophiles*.
